# Measurement in opaque flows: a review of measurement techniques for dispersed multiphase flows

**DOI:** 10.1007/s00707-020-02683-x

**Published:** 2020-05-13

**Authors:** Christian Poelma

**Affiliations:** Multiphase Systems (3ME-P&E), Delft University of Technology, Leeghwaterstraat 21, 2628 CA Delft, The Netherlands

## Abstract

A review is presented of measurement techniques to characterise dispersed multiphase flows, which are not accessible by means of conventional optical techniques. The main issues that limit the accuracy and effectiveness of optical techniques are briefly discussed: cross-talk, a reduced signal-to-noise ratio, and (biased) data drop-out. Extensions to the standard optical techniques include the use of fluorescent tracers, refractive index matching, ballistic imaging, structured illumination, and optical coherence tomography. As the first non-optical technique, a brief discussion of electrical capacitance tomography is given. While truly non-invasive, it suffers from a low resolving power. Ultrasound-based techniques have rapidly evolved from Doppler-based profiling to recent 2D approaches using feature tracking. The latter is also suitable for time-resolved flow studies. Magnetic resonance velocimetry can provide time-averaged velocity fields in 3D for the continuous phase. Finally, X-ray imaging is demonstrated to be an important tool to quantify local gas fractions. While potentially very powerful, the impact of the techniques will depend on the development of acquisition and measurement protocols for fluid mechanics, rather than for clinical imaging. This requires systematic development, aided by careful validation experiments. As theoretical predictions for multiphase flows are sparse, it is important to formulate standardised ‘benchmark’ flows to enable this validation.

## Introduction

Dispersed multiphase flows are encountered in an abundance of industrial processes and natural phenomena, with examples including catalysts in chemical reactors, the production of food, sediment transport in rivers, and blood flow [1]. Dispersed multiphase systems consist of small (typically between $1 \, \upmu$m and a few mm) particles, bubbles, or droplets suspended in a continuous medium. The continuous medium generally acts as a carrier, often exhibiting turbulent flow; the dispersed phase can in turn affect the behaviour of this carrier flow [2].

Despite the fact that multiphase flows are ubiquitous, they remain difficult to predict. While theoretically possible, in practical applications the sheer number of dispersed particles prevents exact numerical solution of the governing equations. Only relatively simple geometries with a limited amount of particles or droplets can be simulated in a fully resolved manner [3]. Current state-of-the-art direct numerical simulations may model up to hundreds of thousands of particles [4]. This is far removed from what is needed to simulate, for example, a human heart pumping approximately $5\times 10^{11}$ red blood cells with each beat. Naturally, detailed knowledge of each of the particles or elements is often not needed, as long as the overall behaviour of the flow is captured. To predict practical applications with realistic computational efforts, one therefore uses models describing the average effects of groups of particles [5]. These models obviously require a thorough knowledge of the underlying physics. Unfortunately, the formulated models are difficult to validate due to a lack of experimental data.

Experimental data sets in multiphase systems are difficult to obtain, as the current state-of-the-art flow measurement techniques used by the fluid mechanics community are based on optical principles—using lasers, cameras, etc. [6]. As soon as there is an appreciable volume fraction of a dispersed phase (as low as 0.5%), the turbidity of the flow renders these techniques useless [7–9].

In this review article, an overview is presented of several (relatively new) measurement techniques that are able to measure in opaque flows, i.e. dispersed multiphase flows that defy quantification using optical techniques. Note that optical flow measurement techniques require not just a transparent *fluid*, but also a distortion-free, transparent *geometry*. The latter is usually referred to as ‘optical access’. Examples where this is an issue include flow measurements inside the human body, turbomachinery, and internal combustion engines. The focus of this review is on techniques that deal with the opacity of the fluid itself, but the techniques can often also be used to address the optical access issue. Flow visualisation and measurement are discussed, with an emphasis on non-intrusive, whole-field measurements, rather than single-point measurements using intrusive probes [10, 11]. Similarly, techniques based on the tracking of individual, labelled particles (e.g. radioactive [12], or magnetic [13]) are excluded. The latter are Lagrangian methods which produce relatively sparse velocity data, often in three dimensions. In principle they *can* also provide time-averaged whole-field velocity data, provided that the experiment can be operated at constant conditions for a sufficiently long time. The focus of this review will predominantly be on measuring velocities and phase distributions in non-colloidal systems. As this manuscript intends to give a broad overview, references are included to more extensive texts or reviews for each of the techniques discussed. Dedicated earlier reviews are available for particular application areas, such as for fluidised beds [14], granular mixing, [15] and chemical reactors [7].

Before addressing the individual techniques, Sect. 2 first discusses some important concerns that are applicable to virtually all measurement techniques. In Sect. 3, special optical techniques are discussed; these can be seen as extensions of the techniques routinely used in single-phase flows. Section 4 briefly discusses electrical capacitance tomography. Three techniques that have risen to prominence in the medical world are discussed in Sects. 5, 6, and 7: ultrasound velocimetry, magnetic resonance velocimetry, and X-ray tomography. The review concludes with a summary and suggestions for future research.

## General issues while measuring in multiphase flows

In this Section, some issues are discussed that either make it difficult to *obtain* data or which make it difficult to *interpret* the data. Some of them might be well known, while others are often overlooked. It has not been the intention to list examples of studies that are flawed (e.g. as they contain cross-talk or might have biased statistics). On the contrary, they are all excellent, state-of-the-art studies that are cited here to demonstrate certain measurement difficulties that might arise in multiphase experiments.

### Cross-talk

Experiments aim at obtaining velocity data of the dispersed phase, the continuous phase, or both simultaneously. In any case, it is essential to know which phase contributed to the information that is acquired. Many studies have used some sort of phase discrimination as a post-processing step of the collected data. In laser Doppler anemometry (LDA), the shape of individual bursts can be used to distinguish between small tracer particles and larger, dispersed phase particles or droplets [16]. Similarly, features in particle image velocimetry (PIV) images can be used to discriminate between fluid tracers and suspended particles. For example, Khalitov et al. segmented images into a series just containing tracer particles and a series just containing dispersed particles, based on the brightness and size of the objects in the image [17]. These two new sets of images were then processed using particle image velocimetry and particle tracking to obtain the velocity fields of the continuous and dispersed phase, respectively. If there is an appreciable mean velocity difference between the two phases, this slip velocity can also be used to separate the two phases [18]. The advantage of these post-processing approaches is that both phases can be obtained with a single instrument (LDA system, camera, etc.). This also guarantees that the data are free from misregistration errors, i.e. errors caused by non-overlapping measurement volumes.

Unfortunately, it is difficult to perform such a phase discrimination with 100% accuracy in this approach. Small differences in particle size, non-uniform illumination, overlapping particles, occlusion by other particles, etc., will lead to variations in the signal. When information of one phase inadvertently spills over into the phase under investigation, this is referred to as ‘cross-talk’. Naturally, this should be avoided, as it makes interpretation of the data very difficult. One approach is to make the selection criteria very strict, avoiding any ambiguous signals. For instance, one can only select very bright and large particles. However, such a strict selection may bias the results, as velocities will predominantly be obtained from, for example, the largest droplets only. These effects can largely be avoided by performing the discrimination not as a post-processing step, but by optically separating the signals before they reach the camera. Later, in Sect. 3.1, examples are given how this can be achieved.

### Reduced signal-to-noise ratio

**Fig. 1:**
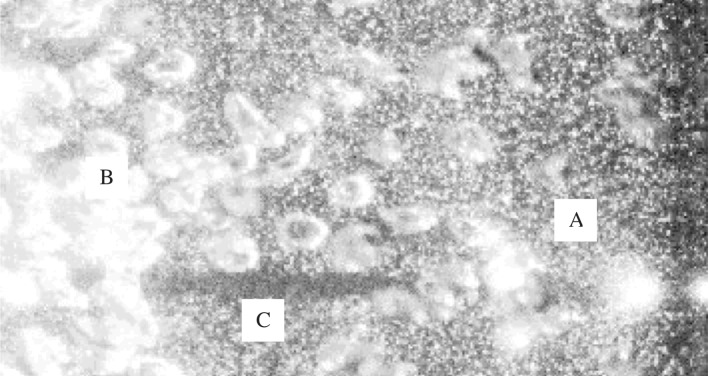
Raw image of a bubbly flow; reproduced from Deen et al. [20] with permission from the authors. The small bright spots are tracers for the continuous phase (water). The presence of bubbles leads to a deterioration of the image, as they block the optical and illumination paths. The area labelled with ‘A’ represents a region with a relatively low bubble concentration, while ‘B’ represents an area with a higher concentration. Above label ‘C’ a shadow can be seen, caused by a bubble inside the light sheet. Note that the majority of the bubbles is outside the light sheet

The presence of a dispersed phase generally reduces the signal quality compared to the single-phase case. For imaging-based approaches, the dispersed phase blocks incoming light from a laser or another source of illumination. For small particles (or bubbles, etc.), scattering leads to a gradual, Lambert–Beer-like exponential decay of the intensity as we move into the medium [19]. This is, for instance, the case when particles are much smaller than the thickness of the light sheet in a PIV experiment. Larger particles or bubbles can create bright specular reflections, overpowering the rest of the image and potentially damaging hardware. These larger structures can create distinct shadows, as can be observed in the region marked by ‘C’ in Fig. 1. This Figure shows a typical raw image taken during a PIV experiment in a bubbly flow [20].

**Fig. 2:**
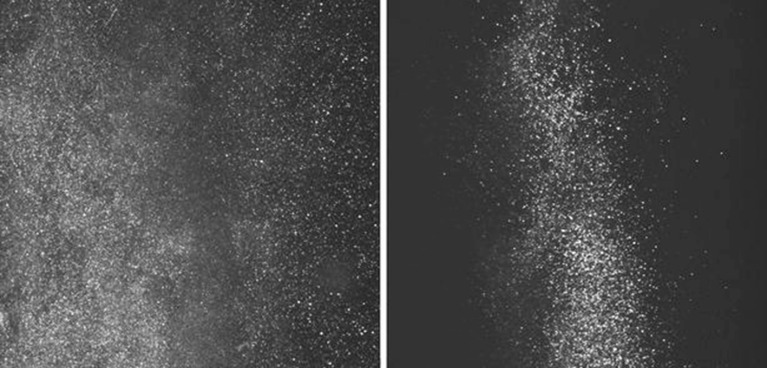
Simultaneous imaging of tracers in the ambient gas phase (left) and spray droplets (right) using two different fluorescent dyes and two cameras with suitable optical filters. Note the dark region in the middle of the left panel, caused by the presence of the jet. Reproduced from Kosiwczuk et al. [26] with permission ©2005 Springer Nature

Even if sufficient light remains, the dispersed elements in between the field of view and the camera (or another detection device) will reduce the signal-to-noise ratio. Depending on the conditions, particles or bubbles may appear as black outlines (i.e. simply occluding the field of view; see, for example, [21]). However, generally they will be out of focus, leading to a blurred, low-contrast image [22]. An example of the latter can be observed in the centre region of the left panel of Fig. 2. The reduced signal-to-noise ratio of these dispersed systems will lead to either noisier velocity data (potentially useable) or will not yield velocity data at all (data drop-out/loss).

### Biased statistics

Under certain conditions, it may seem feasible to accept the data drop-out due to the reduced signal-to-noise ratio and simply compensate for it by collecting a very large amount of data. For example, in a bubbly column some parts may still give a patch of vectors in a PIV measurement or a short burst of LDA velocity measurements. In the example of Fig. 1, some parts of this image (region A) will give a relatively high chance of obtaining useful data. However, these will likely be regions with only a few bubbles, as these conditions lead to a better signal. Regions containing more bubbles may not produce valid data (region B). This implies that the average statistics will not represent the true temporal average of the flow, but will be conditionally averaged, biased towards regions with a lower local concentration of the dispersed phase. To avoid this issue, it is essential to verify beforehand that all events have an equal likelihood of a successful measurement. This is especially the case for bubbly flows, particle-laden jets, sprays, and other flows with considerable concentration variations (e.g. turbidity currents). Even if the data drop-out is truly random (i.e. unbiased), further analysis has to be done with great care if large patches are missing. For instance, if correlation functions or power spectra are of interest, interpolation of the missing data should be avoided as this strongly affects the resulting spectra [9]. Alternative methods, such as ‘slotting’, are more suitable for data with significant drop-out [9, 23].

### Noise level versus turbulence

Even if no signal drop-out occurs, data will generally be noisier due to the presence of a dispersed phase: the image contrast will be lower in PIV recordings, or the shape of bursts will be less than ideal in LDA data. Both lead to increased (random) errors in the estimation of the velocity. Depending on which information is of interest, this can in turn lead to erroneous flow statistics. A very thorough analysis is given by Adrian and Westerweel [24]. While that monograph deals with PIV, the theory is readily applicable to other measurement techniques. Only a brief summary is given here:

Assume a true, instantaneous velocity *u* and a random measurement error $\epsilon$ (ignoring systematic errors). A measured velocity will thus be ${\hat{u}}=u+\epsilon$.

When estimating the mean velocity *U*, the random error cancels out, so that the correct mean is estimated:

1 $$\begin{aligned} U=\langle {\hat{u}}\rangle =\langle u+\epsilon \rangle = \langle u\rangle +0=\langle u \rangle \end{aligned}$$

However, if we want to estimate the variance $\sigma$ (e.g. to estimate the turbulent kinetic energy), we find:

2 $$\begin{aligned} \sigma =\langle {\hat{u}}^2\rangle = \langle (u+\epsilon )^2\rangle = \langle u^2\rangle +\langle \epsilon ^2\rangle + \langle 2 u \epsilon \rangle . \end{aligned}$$

Note that, for simplicity, the mean value is not subtracted. The last term is zero if the error is uncorrelated with the actual velocity value (which is generally the case). However, the average of the *error squared* is nonzero. Hence, the measured variance will be the sum of the contribution from the actual velocity fluctuations and from random errors. While for most well-designed single-phase experiments the error in the velocity is in the order of 1%, this error can increase considerably in multiphase experiments. For example, error contributions to the uncorrected variance of up to 25% have been reported [9]. If subtle differences compared to a reference single-phase flow are investigated, it is essential to quantify the noise level before a meaningful comparison can be done. One way to achieve this is by evaluating the correlation function of the velocities, either using spatial or temporal data series. Even without knowledge of the expected shape of the correlation function, this method allows one to separate correlated (physical) and uncorrelated (noise) parts of the signal. This approach is better than blindly ‘smoothing’ the data, yet it requires sufficient spatial or temporal resolution [25].

## Optical approaches

Over the years, there have been many approaches to expand the capabilities of existing optical techniques in densely laden flows. In this Section, a number of common approaches are reviewed that stretch the limits of imaging-based techniques.

### Fluorescent tracers

A number of problems discussed in Sect. 2 can be reduced by using *fluorescent tracers*, rather than conventional tracer material for the continuous phase. This concepts relies on the fact that these small tracers will emit light at a different (longer) wavelength than the (laser) light used to illuminate the field of view. The advantage is that only the tracer particles will be visible on a camera equipped with an appropriate band-pass filter to block the original laser light. Scattering and reflections of the geometry, bubbles, or dispersed phase particles are effectively removed, which allows measurement at somewhat higher volume fractions, as the imaging conditions (aperture, exposure time, etc.) can be optimised for just the fluorescent particle images. Recently, a variant of the fluorescent tracer approach was introduced that utilises the *incandescence* of small tracer particles, heated by a laser light sheet. The larger droplets that formed the dispersed phase were not heated significantly, yet the small, tungsten carbide tracer particles created a signal that could be separated from the scattering of the dispersed phase [27]. These approaches, based on wavelength separation, also no longer require image processing to distinguish between tracer and dispersed phase particles. This strongly reduces chances of cross-talk (see Sect. 2.1). Unfortunately, the presence of a dispersed phase will still lead to the same reduction in image quality, so that attainable volume fractions are still modest.

As a camera with a band-pass filter will only record the fluorescent light of the tracers, a second camera is needed if the dispersed phase is also of interest. Often the scattering and fluorescence intensity of the tracer particles is weak enough so that their signal is comparable to the noise level of the camera. This implies that no filters are needed for the camera that images the dispersed phase [9]. For small particles or droplets (comparable to the tracer material), more elaborate approaches need to be used to avoid any possible cross-talk. An example is the use of two different dyes, with an appropriate band-pass filter on each camera [28]. If only mean statistics are of interest, two experiments can be performed in succession to obtain each of the phases sequentially (with and without filter). Naturally, only simultaneous recording using two cameras will provide instantaneous velocity data, significantly facilitating the study of the interaction of the phases.

Fluorescent tracers have been used to study both phases in bubbly flows [8, 21], particle-laden flows [9, 29], and sprays [26, 30]. These examples show that the technique works for any combination of continuous phase (gas/liquid) and dispersed phase (gas/liquid/solid). An example of raw image data obtained using two different fluorescent dyes in a spray is shown in Fig. 2. Note that the costs of commercial fluorescent tracers can be prohibitive for large-scale experiments, but methods to produce them in-house are available [31, 32].

### Refractive index matching

The deterioration of the image quality in multiphase flows, as described in Sect. 2.2, is caused by scattering and refraction of light in and around the field of view. These effects are governed by the difference in the index of refraction of the continuous and dispersed phase. By selecting two phases with identical indices, the flow becomes transparent, as light propagates unhindered and the dispersed phase ‘disappears’. This approach is also used to measure in fluids that are transparent, but which flow in devices that have a complex geometry with limited optical access. By constructing geometries from materials that have the same refractive index as the fluid, detailed measurements are possible, even through strongly curved walls. Examples include studies inside a cylinder head of an internal combustion engine [33], turbomachinery [34, 35], blood vessels [36, 37], and nasal cavities [37, 38]. In some cases, such as the flow through a packed bed (discussed later), the distinction between the ‘opacity of the suspension’ and ‘limited optical access due to the geometry’ becomes blurred. As the dispersed phase disappears completely in a well-matched system, a small subset of the dispersed particles is often marked (e.g. dyed) to be able to obtain velocity information without affecting the image quality too much [39]. For large particles, it is also possible to embed fluorescent marker dots on the surface of the transparent sphere, so that the particle velocity *and* rotation can be tracked [40].

Refractive index matching has been demonstrated first in the 1990s for LDA [41] and PIV [42]. As the refractive indices of gases under normal conditions are always much lower than those of liquids and solids, it is not feasible to perform index matching in gas–liquid systems. All studies using refractive index matching thus focus on liquid–liquid or solid–liquid systems. Reviews of suitable combinations are given by Budwig et al. [41] and more recently by Wiederseiner et al. [43] and Wright et al. [44]. Unfortunately, finding suitable combinations for the materials leads to a rather restrictive parameter space: the density and viscosity ratios cannot be chosen freely, as one has to rely on materials that have relatively similar physical properties. Furthermore, the materials should be available at reasonable cost, but also have limited corrosiveness and toxicity.

**Fig. 3:**
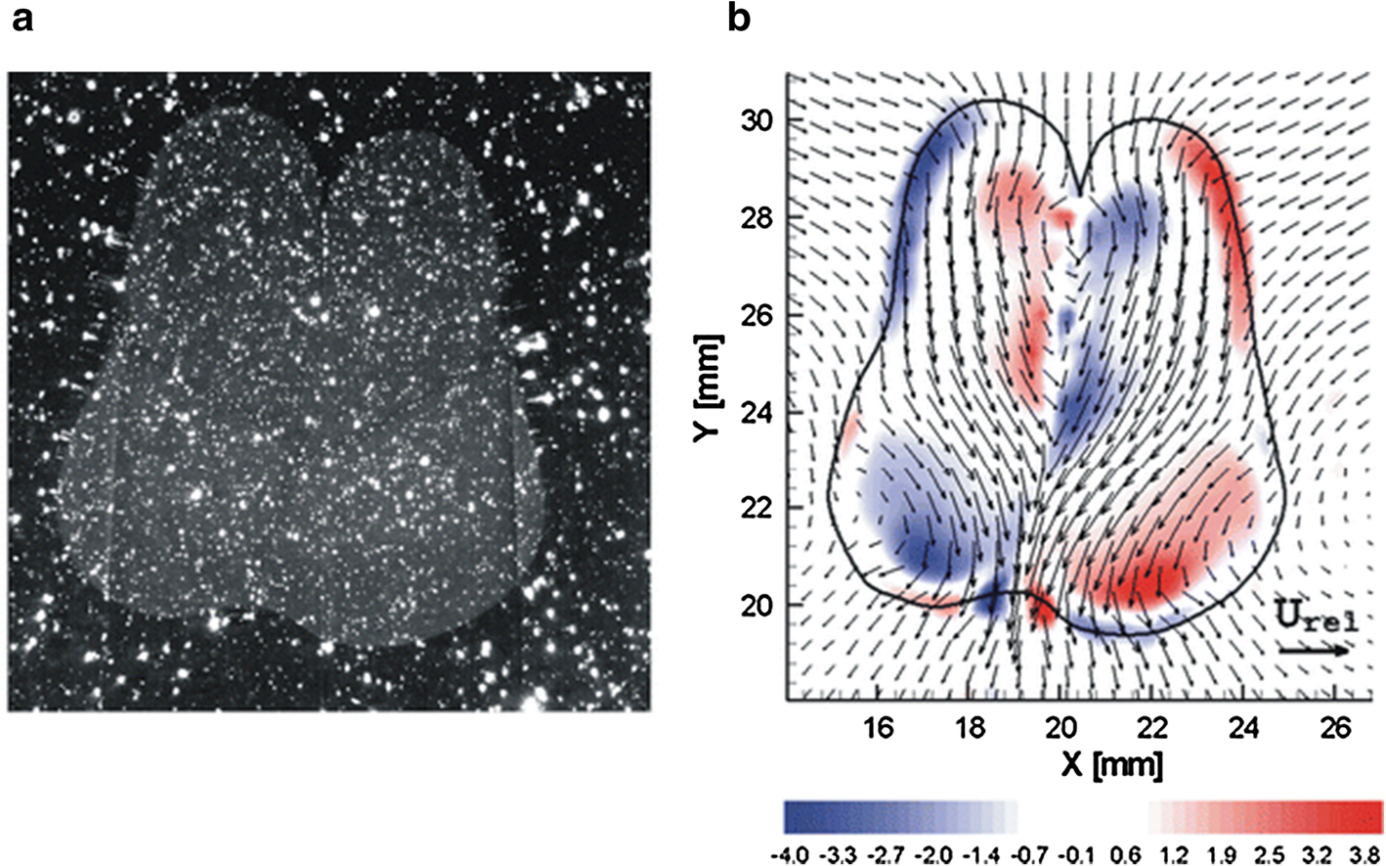
Droplet collision study using refractive index matching of both liquids, to allow simultaneous velocity measurement outside and inside the droplets. Panel *a* shows the raw images (notice the seeding particles in both phases and the fluorescent dye to demarcate the droplet phase). Panel *b* shows the velocity field (vectors) and the vorticity (colour coding). Reproduced from Kim and Longmire [45] with permission ©2009 Springer Nature (color figure online)

Examples where the technique has been shown to be useful include studies of droplet–droplet interaction [45, 46] and film rupture [47] in liquid–liquid systems. An example of a PIV measurement to study droplet–droplet interaction is shown in Fig. 3, where two droplets have just coalesced. The strong curvature of the interfaces would lead to strong distortion without index matching, making visualisation of, for instance, film drainage near-impossible.

While the idea is simple, the practical implementation of the refractive index matching technique can be difficult. Refractive indices are dependent on temperature [48]. Similarly, evaporation will affect the refractive index of mixtures or salt solutions used as continuous phase [49]. It is difficult to give precise guidelines for the required agreement of the refractive indices to obtain accurate data. For systems that are geometrically simple, such as, for example, a Taylor bubble, it is possible to use ray tracing to predict the exact distortion or refraction due to a mismatch in the refractive index [50]. Dijksman et al. performed ray tracing to generate images through stacked layers of spheres with a small mismatch. They derived an acceptable upper limit of the mismatch of 0.2% for their application [51]. For systems containing many small, dispersed particles or droplets, this ray tracing approach is no longer feasible. For relatively large objects, examples are available where a 0.5% mismatch in the refractive index made the difference between a completely transparent system and one where the contours of the solid phase were visible [49]. This implies that the refractive indices need to match the third decimal, requiring precise temperature control of the experiment. A further complication is the fact that commonly used dispersed phase material is often produced in bulk. Non-uniformities in the refractive index can be introduced during manufacturing of glass beads, which will limit the success of index matching [52]. Between batches of the same particles, variations in the refractive index of 0.01 (or 0.6–0.7%) have been reported [51]. For a given volume fraction, a smaller dispersed phase will result in a larger number of interfaces where slight refraction can occur. Hence, most studies that successfully employ refractive index matching use fairly large dispersed phases. In particular, studies through porous materials or packed beds lend themselves to this approach [48, 53]. Recently, there is an increased interest in hydrogels—particles that absorb water and swell to millimetre-sized spheres [40, 54]. As they consist mostly of water, their refractive index is easily matched. Recent examples are particle–flow interaction [55] and flow through a porous medium [56]. A drawback is their lower mechanical strength compared to solid particles.

### Ballistic imaging

Ballistic imaging allows visualisation of densely laden flows with a much improved contrast and clarity [57]. The technique makes use of the (very small) difference in passage time of photons that travel without interactions and photons that scatter at least once, due to droplets or particles in the observation region. To achieve this, the technique requires very short (picosecond) pulsed laser illumination and/or a very fast camera shutter. The fast shutter essentially filters out the scattered photons (which have a longer path and thus time of flight), so that the unscattered photons can be used to create a shadowgraphy image. By removing light that scatters multiple times, an image with a higher contrast can be obtained [57]. Due to the complicated optical set-up and specialised light source, only a few applications have been reported—mostly in sprays, by a limited number of research groups [58, 59]. When the system is expanded to two ultrashort light sources, the velocity field can be determined; see, for instance, the study by Sedarsky [60]. By utilising a third illumination pulse, even the acceleration field can be determined [61]. As stated, this approach requires equipment that is not readily accessible to most researchers, hence it will likely not find widespread use in dispersed multiphase flows in the near future.

### Structured illumination

**Fig. 4:**
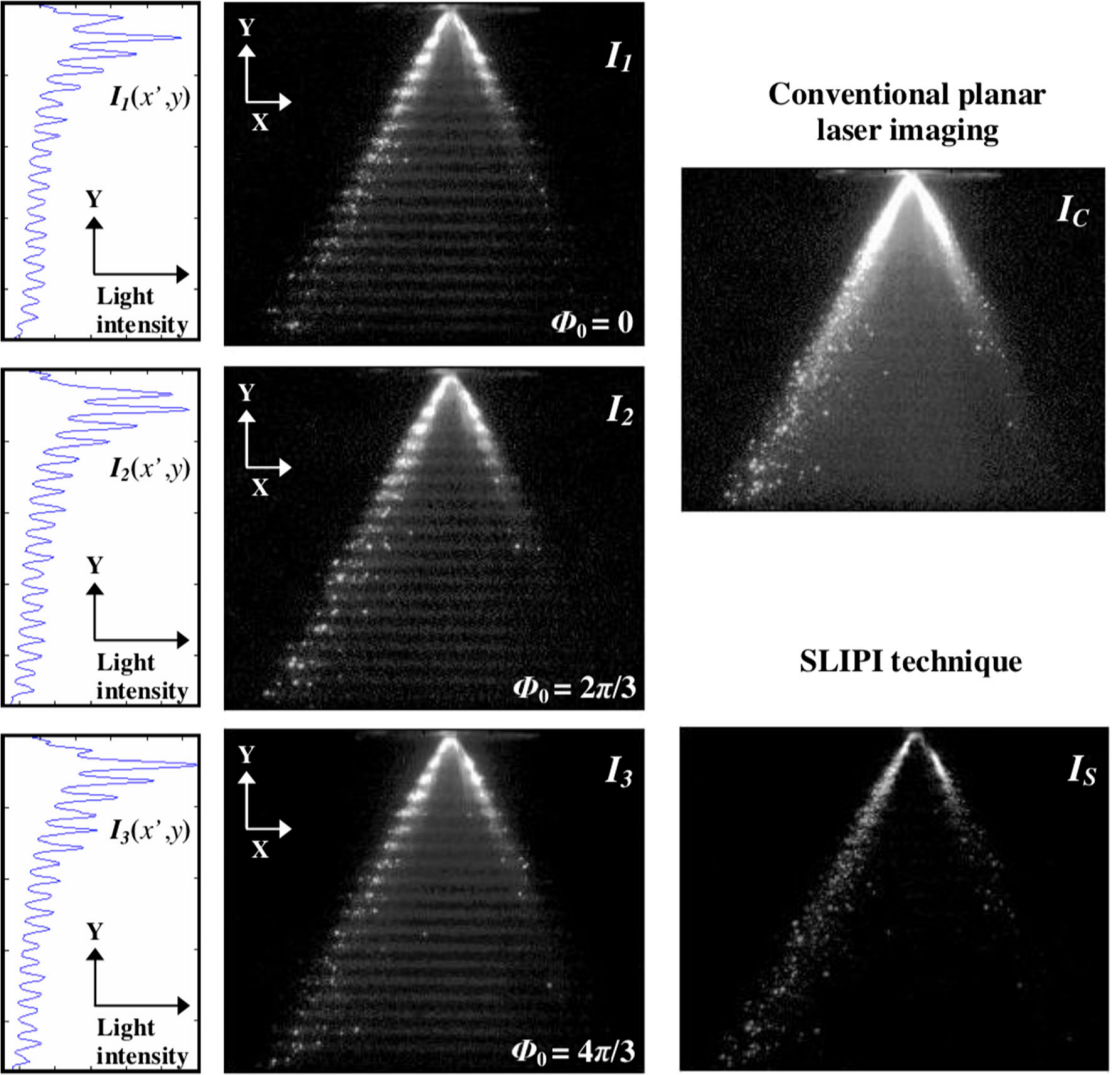
A spray imaged using the structured illumination (SLIPI) method. Three images are obtained using a modulated light sheet, each with a $2\pi /3$ phase difference in the *y*-direction. Adding these will give a conventional image (top right), while the pair-wise difference gives a higher-contrast image, as multiple scattered light is removed (bottom right). Reproduced from Berrocal et al. [63] with permission ©2008 The Optical Society (OSA)

In the previous approach (ballistic imaging), light that scatters multiple times was removed by very fast shutters. An alternative method to remove contributions from multiple scattering is to use *structured illumination*. The main idea of this technique is to create a recognisable signature in the light that scatters from within the laser sheet, while light that scatters multiple times (outside the sheet) loses this signature [62]. In the structured laser illumination planar imaging (SLIPI) method [63], the field of view is therefore not illuminated by a conventional, uniform light sheet. Rather, three consecutive images are recorded with a spatially modulated illumination to create the ‘signature’. As shown in Fig. 4, each illumination is modulated in the *y* direction with a sine wave and there is a shift of $2\pi /3$ between the recordings. If these recordings are simply summed, a conventional image is obtained (as if a uniform light was used); see the top right panel of Fig. 4. However, by calculating the pair-wise difference,^1^ an image can be reconstructed without multiple scattered contributions (the ‘SLIPI’ image, bottom right of Fig. 4).

As with ballistic imaging, the optical set-up is more complex than conventional imaging. Apart from the need for three pulsed illumination sources, an accurate spatial phase shift has be introduced for each illumination. The images need also to be captured sequentially, but within a timeframe so that there is minimal motion of the scatterers. In the original paper by Berrocal et al. a rotating glass plate in combination with a grating was used to create the modulation offset (i.e. phase shift). The total acquisition time for their system was $110\,\upmu$s, which meant that only time-averaged images could be obtained for a spray. For slower flows (< 25 cm/s with their set-up), true ‘single-shot’ images could be obtained [63]. In a more recent paper, a version of the method requiring only two images was presented [64], making the technique more feasible using the common dual-head lasers used for PIV. With two images, residual lines appear in the reconstructed image, which need to be filtered by means of a low-pass filter. By carefully choosing the modulation pattern, this filtering can be done without affecting the image features that are under investigation. The SLIPI method, predominantly applied within the spray and combustion community, has also been extended to obtain droplet diameters and concentrations [65].

### Optical coherence tomography

Optical coherence tomography (OCT) is a low-coherence interferometry method [66] that can be considered to be the optical equivalent of ultrasound imaging (see Sect. 5): an image is reconstructed by scanning a sample and evaluating the reflections (amplitude and time of flight) of a laser beam. While this method can achieve a superior resolution (sub-micrometre), the drawback is a limited depth range (of order millimetres) and slow imaging rate; the latter inhibits correlation-based flow measurement in, for example, turbulent flows. Velocities can be obtained from the Doppler shift in the signal [67], but this will only provide the wall-normal component if the laser beam enters perpendicular to the flow. While most of the applications of OCT have focussed on blood flow measurements, there are some proof-of-principle measurements in (steady-state) particle suspension flows [68]. A recent review by Koponen et al. summarises applications in dispersed multiphase flows [69].

## Electrical capacitance tomography

**Fig. 5:**
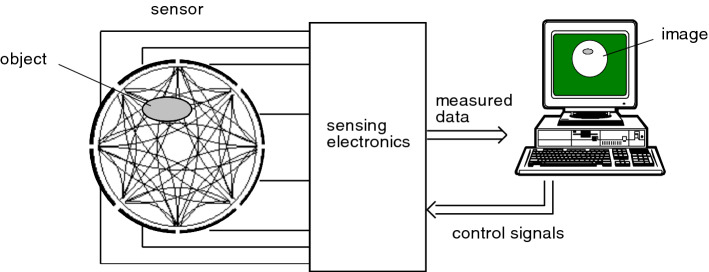
A schematic representation of a typical electrical capacitance tomography system. Reproduced from Yang and Peng [71] ©2002 IOP Publishing. Reproduced with permission

Electrical capacitance tomography (ECT) is a technique that uses the variation of dielectric properties within the measurement region to reconstruct the distribution of two or more phases [70]. It utilises a number of electrodes that are mounted in the wall of (typically) a pipe; see Fig. 5. As a bubble or droplet with different dielectric properties moves through the field of view, it will affect the electrical signal travelling between two electrodes. Typically, a voltage is first applied to one electrode, and the charge on the remaining electrodes is recorded [71]. This process is then repeated sequentially with the other electrodes. The entire process typically takes milliseconds, but this obviously depends on the hardware used [72]. The material distribution is not measured directly, but must be reconstructed by solving an inverse problem using the data obtained from the electrodes [71]. The theoretical resolution of this tomographic reconstruction is proportional to the number of electrode pairs used: for N electrodes, there are $N(N-1)/2$ independent capacity measurements. As generally a relatively small number of electrodes is used (10–20), this means that only large-scale structures can be reconstructed. Small individual particles or bubbles thus cannot be resolved, nor tracked. Simply increasing the number of electrodes is not trivial, as that would reduce their size and thus their sensitivity. Furthermore, it increases the complexity of the electronics hardware and achievable frame rate [72]. Nevertheless, the technique is able to discern flow regimes (‘stratified’ versus ‘churn flow’, etc.). Depending on the distribution of the electrodes, the technique will provide cross-sectional slices or even 3D volumes [73].

Calibration of ECT systems to obtain accurate quantitative data is not trivial [74]. As velocity information is not available and the technique has a relatively low resolution, it is currently suitable for mostly qualitative flow regime classification. Applications and recent developments are summarised in recent review papers. [75, 76]. Note that other application areas use variations of the technique, such as electrical resistivity tomography for geophysical studies [77] and electrical impedance tomography in clinical studies [78].

A related technique is the use of a mesh of conducting wires in the cross section of the pipe. By sequentially measuring the electrical current between combinations of wires, the local permittivity can be determined [79]. With a calibration procedure, this can be used to determine the local void fraction [80]. While no longer a non-intrusive measurement technique, it can give fairly detailed insight in, for example, gas–liquid flows. As the flow passes through the wire mesh, the subsequent slices can be stacked to reconstruct a quasi-3D distribution of both phases [80].

## Ultrasound

Ultrasound-based techniques have been a mainstay in medicine for more than half a century [81]. In its original form, it was solely a technique for *imaging* inside the human body. It creates these images by sending acoustic wave packets (with typical frequencies of 1–10 MHz) through a sample and recording the echoes [82]. The delay in the received signal is determined by the depth at which the echo was created, while the intensity gives information about the local (change) in material properties—in particular the acoustic impedance (density multiplied by the speed of sound). The result is a (near-)instantaneous image representing a relatively thick slice of the region of interest. The stronger the change in impedance, the more of the acoustic wave is reflected. This also implies that the transition from water to air leads to a very strong reflection, with little signal propagating deeper into the system. Many common materials have a relatively similar impedance, so that sound can penetrate—to some extent—through geometries that are not transparent to visible light. Examples include non-transparent elastic tubes [83, 84], PVC pipes [85], and of course tissue [86, 87]. Besides circumventing the ‘optical access’ criterion, sound also penetrates through suspensions, avoiding the opacity issues associated with visible light.

‘Doppler imaging’ (or ‘colour Doppler’) was introduced later, which superimposes the velocity field as a colour map on the original ultrasound image. This velocity, as the name implies, is derived from the Doppler shift in the received echoes. Note that only the component along a single axis—the propagation direction of the sound waves—can be obtained.

These clinical techniques also found their way to fluid mechanics research. An extensive review of ultrasound-related applications and developments is available elsewhere [88]. In short, three techniques are in use: time-of-flight flow metering, Doppler profiling, and correlation-based techniques. The first of these provides only a mean flow rate [89] and is thus not very useful for most research situations. It will therefore not be discussed here.

### Doppler profiling

Doppler profiling uses a single line (in contrast to the imaging discussed above) to estimate a velocity profile. As the Doppler shift is only caused by velocities in the direction of the propagation of sound, the axis is generally placed at an angle to estimate the velocity profile in a pipe. By selecting different delay ‘windows’ for the received echoes, the velocity at different depths can be obtained. The technique was made popular outside clinical settings by Takeda in [90], who also popularised the term ‘ultrasound velocity profiling’ (UVP) [91]. It is a popular technique in hydrological studies [92], as it does not need calibration, can extract velocity and density information over a large profile, and can be housed in a single, sturdy instrument to be used in the field. Other application areas include food production [93] and pulp flow during the production of paper [94]. Unlike time-of-flight measurements, Doppler profiling (and imaging) requires changes in material properties to create echoes. It thus cannot work in a single, continuous medium (e.g. clear water). However, as these techniques are deployed exactly *because* of the opacity of a fluid, this is obviously not an issue.

### Ultrasound imaging velocimetry (‘echo-PIV’)

Already in the late 1980s, the idea was pitched to estimate flow velocities using the correlation of features in ultrasound images [95]. The technique was introduced to measure blood flow in clinical settings, but never gained traction. In the early 2000s, it resurfaced in the fluid mechanics community by the work of Kim et al. [96]. Several names are used for very similar measurement techniques: digital ultrasound speckle imaging velocimetry [97], ultrasound imaging velocimetry (UIV) [88] and echo-PIV [96]. The latter name invokes the basic concept: images are acquired with an echography system (rather than a camera), and these images are processed using conventional PIV algorithms to estimate the local velocity. While this explanation conveys the general approach—and is accurate with respect to how the first studies were performed—it ignores many of the intricate differences between conventional PIV and ultrasound-based PIV. One important example is the sweep correction that is needed due to the fact that the ultrasound image is not obtained instantaneously, but in a column-by-column fashion. This means that velocities (and accelerations) need to be corrected to account for the time difference between various regions of the image [98]. Another big difference is the large resolution difference in the axial and transverse direction (i.e. ‘horizontal’ and ‘vertical’ axis of the image), as well as the way the image is produced from the raw data. These raw data, the output of piezo-electric transducer elements, are traditionally processed to generate a clean, noiseless image for clinical evaluation. This leads to processing steps that are generally not optimised for correlation-based velocity estimates (e.g. de-noising, lossy compression) [88]. After a decade of mostly development and validation studies, the technique is currently employed for, among others, in vivo blood flow studies [87, 99] and fundamental studies of particle-laden flows [100].

**Fig. 6:**
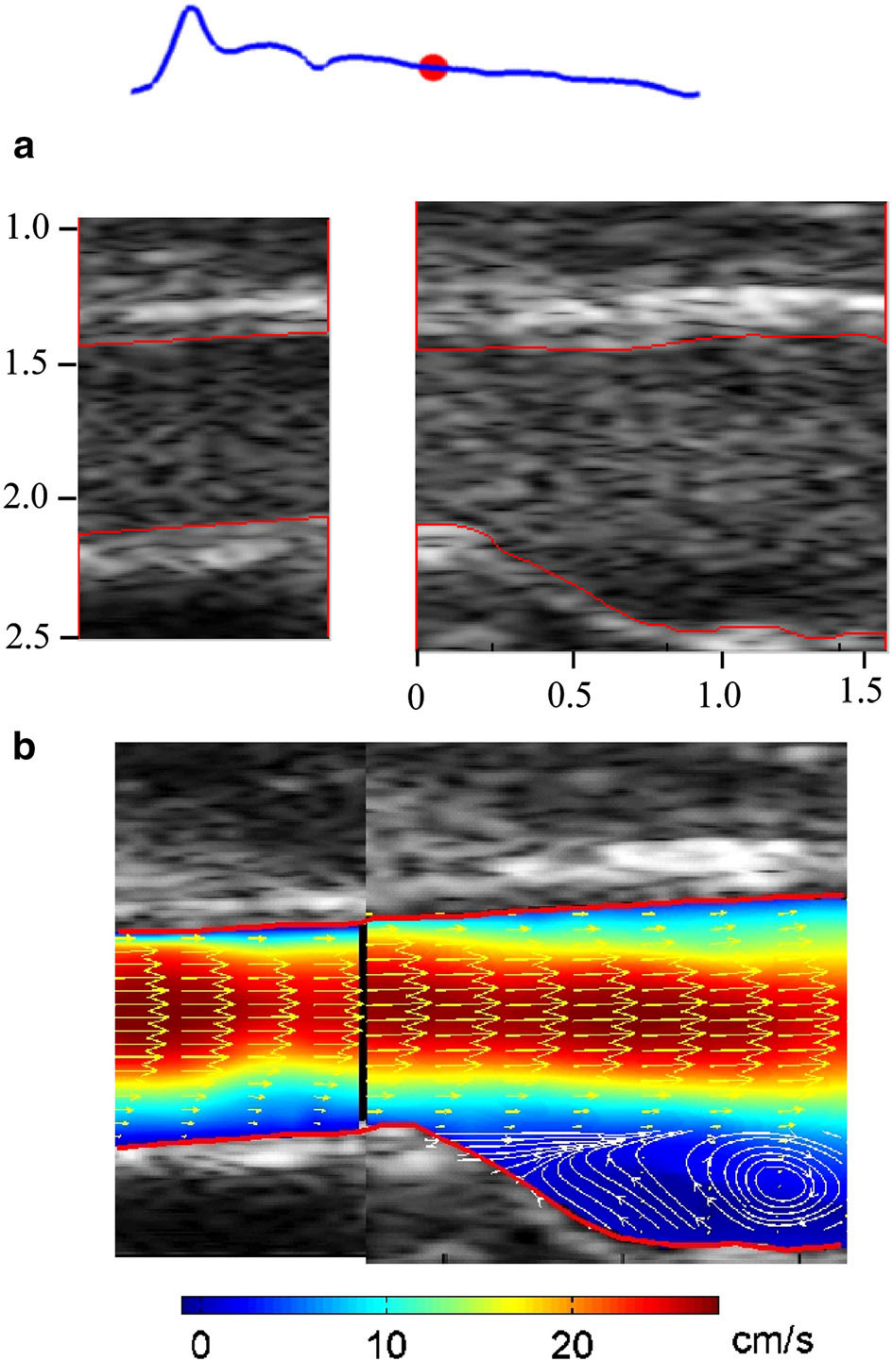
In vivo measurement of the velocity in a carotid artery using ultrasound imaging velocimetry/echo-PIV **a** raw ultrasound images (using contrast medium). The tick marks have units of centimetres, **b** instantaneous velocity field, combining data from two measurements. The colours represent the velocity magnitude. The red marker at the top indicates the time instance during the cardiac cycle (blue curve). Reproduced from Zhang et al. [86] with permission ©2011 Elsevier (color figure online)

As ultrasound images are created based on changes in acoustic impedance, it is generally the dispersed phase that will create the signal. As such, the outcome of UIV is the velocity field of the dispersed phase. For colloidal particles (much smaller than the wavelength of sound), the signal will be speckle-like in nature [101]. For larger particles, the individual particle images can often be distinguished in dilute flows [102]. As water by itself will not create a signal, tracer material has to be added for single-phase measurements. While UIV might not be the first choice for single-phase measurements, it is common to perform reference measurements with the same technique before addressing particle-laden cases [100]. For single-phase measurements, conventional PIV tracer material can be used [102]. Much higher signal-to-noise ratios can be obtained by using tracer material specifically designed for ultrasound imaging: contrast medium. Although pricey, this ‘contrast-enhanced’ echo-PIV approach provides unsurpassed image (and thus velocity) information [86, 87]. An example of a contrast-enhanced UIV measurement of the velocity field in a carotid artery is shown in Fig. 6.

**Fig. 7:**
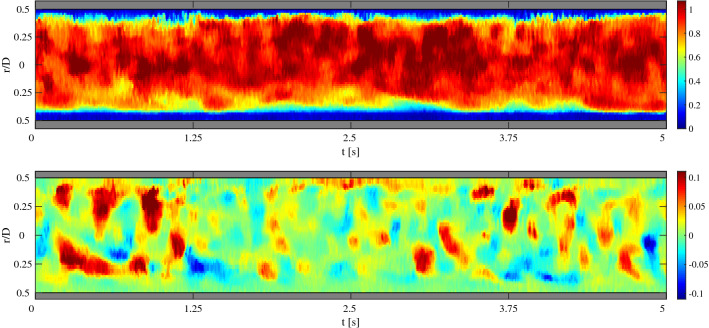
Axial (top) and radial (bottom) velocities in a fully developed turbulent pipe flow (Re = 5300), obtained with UIV using plane-wave imaging at 1 kHz. From each vector field, one radial profile is shown as a function of time. Reproduced from Hogendoorn and Poelma [25]

The two main drawbacks of UIV are the relatively low resolution and the maximum achievable frame rate. The low resolution—compared to optical techniques—directly follows from the much longer wavelength of ultrasound. For most studies the image resolution is 0.1–0.5 mm and the resolution of the velocity field is often an order of magnitude coarser. While the use of higher ultrasound frequencies (and thus shorter wavelengths) can improve the resolution, it leads to a reduced signal penetration depth [88], as sound is absorbed more at higher frequencies. The maximum achievable frame rate predominantly stems from the relatively low speed of sound: in conventional ultrasound imaging, the time required for the sound wave to travel through the sample and back leads to a non-negligible delay time. As typically 128–256 columns are read-out sequentially, the frame rate is often limited to 50–200 images/second. This limits the velocities that can be measured: for very fast flows, image features will have left the image in the subsequent frame. The frame rate can be improved by using ‘plane wave imaging’, where all transducer elements emit and are read-out at the same time, rather than sequentially [103]. While allowing frame rates of several thousand images per second, it leads to a lower signal-to-noise ratio [25]. Nevertheless, this technique has been a breakthrough, allowing time-resolved velocity measurements. An example is given in Fig. 7, showing data of a turbulent pipe flow obtained at 1 kHz. Recently, even 4D measurements have been reported [104]. These measurements relied on a prototype matrix transducer (rather than the usual linear transducer), which are not yet readily available. Furthermore, averaging over several cycles was needed to obtain a sufficient signal-to-noise ratio.

## Magnetic resonance imaging

After breakthrough developments in the late 1970s, magnetic resonance imaging (MRI, previously known as nuclear magnetic resonance) became a powerful clinical imaging modality in the 1980s [105]. It enables visualising in non-transparent media, with the human body being the principle application. Similar to ultrasound, it both addresses the ‘optical access’ issue, as well as the opacity of the fluid.

In MRI, a strong permanent magnetic field is used to align the magnetic moment of (typically) hydrogen nuclei, i.e. protons [106, 107]. This equilibrium state is then perturbed by a radiofrequency pulse. After this perturbation, the protons will return to the equilibrium state, which creates a magnetic flux that can be detected by receiving coils outside the measurement domain. The relaxation response of a proton is determined by its bonding state, so that the relaxation time can be used to distinguish various compounds. As protons respond only to certain frequencies (which are in turn dependent on the applied magnetic field strength), the use of linear gradients in the magnetic field can be exploited to image only a certain region in space. To devise acquisition strategies and to process the collected data, it is convenient to switch from 2D or 3D images to their Fourier-transformed counterparts; this is referred to as ‘k-space’ [106].

While originally an imaging technique, it was extended to blood flow measurement in the mid-1980s [108]. This was achieved by using two gradient pulses with opposite phase. For non-moving material, there will not be a phase shift in the recorded signal. If material moves between the two pulses, a phase shift can be observed. This approach is often referred to as phase-contrast MRI.

An early review of the possibilities of MRI for general fluid mechanics is presented by Fukushima [109]. More recent reviews are given by Elkins et al. [110] and Gladden et al. [111], while Stannarius [112] summarises MRI applications in granular matter. When the main goal is to obtain flow fields (rather than imaging), the technique is often referred to as ‘flow MRI’ [111] or magnetic resonance velocimetry (MRV) [110].

One of the first more rigorous assessments of the capabilities for flow measurement was done by Ku et al. [113]. They used, among others, fully developed pipe flow and the flow through a curved pipe to investigate the suitability of the technique to quantify flow separation and turbulence. They reported a deterioration of the results at higher turbulence levels. This could in part be attributed to vortical structures smaller than the voxel size. Furthermore, fluctuations that are faster than the response time may provide random results [113]. This response time, as discussed above, is dependent on the state of the protons. As water has a long relaxation time, it is often doped to strongly reduce the relaxation time. A typical image acquisition collects signal during a timescale of the same order as this relaxation time. This means that acquisition times of doped media are shorter, which improves the frame rate in so-called cine MRI (time series of MRI images). It also vastly improves the image quality due to the fact that the signals are now emitted in a shorter time. Common doping agents are $\text {MgCl}_2$ [113], gadolinium-based [114], or $\text {CuSO}_4$ [115].

Within the general fluid mechanics community, research groups in Cambridge [116, 117] and Stanford [118, 119] paved the way. The latter closely collaborated with the group in Freiburg [120, 121]. In Stanford, the technique was extended to also measure concentration [122] and temperature fields [123]. In Cambridge, the technique was used to study the flow in packed beds [124], fluidised beds [125], and to investigate turbulence created by rising bubbles [126]. While these studies predominantly utilised MRV to deal with the opacity of the fluid, it has also been used to measure in (transparent) fluids in complicated geometries. Examples include the intake of an IC engine [127], stenotic blood vessels [128], model aneurysms [129], and nuclear fuel rod bundles [130].

Time-dependent measurements, such as the study of bubble-induced turbulence [126], are made possible by means of so-called ultrafast MRI, which allows recording velocity fields in a single slice up to 100–200 times per second [111]. It should be noted that the famous ‘4D’ visualisations of blood flow [131] are based on phase-averaged recordings collected over many minutes and do not represent instantaneous data [132]. There is a continuous drive towards faster acquisition times, by new hardware and acquisition strategies, to better investigate unsteady phenomena such as turbulent flows. For instance, the use of compressed sensing allows a dramatic reduction in the acquisition time [115]. In a recent study, a ‘4D’ data set of a cardiovascular flow could be obtained within 2 min [114].

**Fig. 8:**
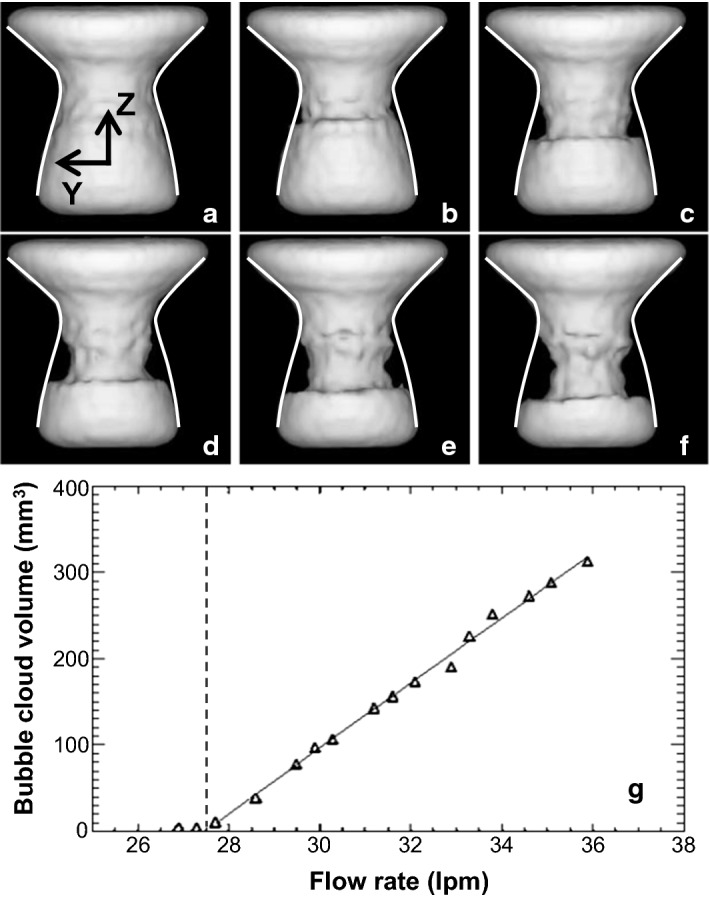
Reconstruction of the time-averaged liquid and vapour phase distribution in a cavitating venturi. The venturi geometry is indicated by the white contours and the flow is from the top to bottom. Case **a** is non-cavitating, showing only liquid (white isosurface). Case **b**–**f** shows that with increasing flow rate there is a larger vapour region (black region just below the throat). The bottom figure shows a quantitative analysis of the volume of the cavitation cloud as function of the flow rate. Reproduced from Adair et al. [134] with permission ©2018 Elsevier

**Fig. 9:**
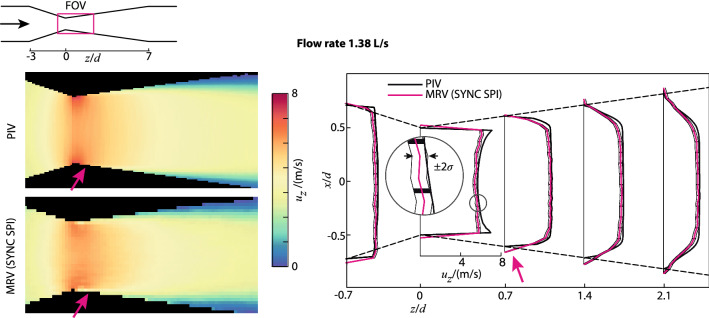
Average velocity field in a venturi (non-cavitating case). Validation of MRV (bottom left) against PIV reference data (top left). The right-hand side figure shows that in regions with strong gradients the MRV data is smeared out compared to the reference PIV data. Reproduced from John et al. [115] with permission ©2020 Springer

To demonstrate the capabilities of MRV in opaque flows, two examples are shown here of its application to the flow through a venturi (with the aim to investigate cavitation). Recently, it was proposed that the technique can also quantify void fractions in, for example, cavitating flows [133, 134]. Figure 8 shows 3D reconstructions of the cavitation cloud in a venturi. Figure 9 shows the velocity field of the continuous (water) phase in a different, but very similar experiment using a venturi. In this study a comparison was made with reference data using PIV. The agreement was good, apart from a spatial averaging effect in the regions with a strong spatial gradient downstream of the throat.

Typical resolutions of MRI-derived flow fields are of the order of 0.2–1.0 mm (see, for example, Table 1 of [115]). Note that each voxel here contains one velocity estimate. This is in contrast to UIV, where the velocity is estimated using a group of pixels in an interrogation area. Due to the use of strong magnetic fields (0.5–3 T), ferromagnetic materials cannot be used in the vicinity of the MRI device. Non-ferromagnetic materials can in principle be used, but they distort the magnetic field and thus affect the measurement result if no correction is applied. With the rapid rise of additive manufacturing techniques, complex geometries can be created using MRV-compatible materials [127–130]. As MRI devices are expensive to acquire and to operate, only a limited number of research groups have dedicated facilities. Most experiments are performed in close collaboration with medical centres.

Similar to the process of translating ultrasound from clinical applications to fluid mechanics research, a number of limitations needed to be overcome for MRV. One of them is the occurrence of misregistration errors that appear in fast flows, i.e. flows that exceed the velocities of maximally 1 m/s seen in cardiovascular flows. These arise from the fact that the linear gradients and read-out are not simultaneous—the fluid travels between various encoding events. Recent work has highlighted these errors and provides an acquisition sequence that strongly reduces them [115]. There are still open questions, such as the effect of turbulence or the presence of a vapour phase on the accuracy of the velocity results. The latter has not been established, mostly because there is very little reference data in these flows.

## X-ray imaging and tomography

**Fig. 10:**
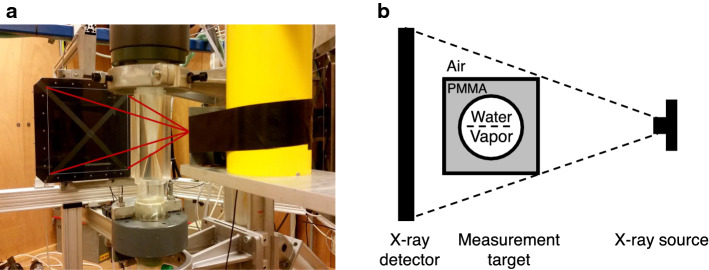
**a** An X-ray computed tomography facility to investigate a cavitating venturi. The yellow device contains the source, the black square is the detector. The venturi is placed in the measurement area in between the source and detector. **b** A schematic representation. Reproduced from Jahangir et al. [144] with permission ©2018 ASME

The third and final modality that is widespread in the medical world and has found its way to fluid mechanics is X-ray imaging [135] . X-ray imaging is a shadowgraphy technique, where a sample is placed between a source and a detector (Fig. 10). X-rays can travel with relatively little absorption through opaque media, compared to visible light. The absorption is proportional to the amount of matter present at a certain location, i.e. the local mass density. X-rays do not significantly refract, as the refractive index is very close to unity. The amount of intensity ‘lost’ at the detector is thus proportional to the integrated densities along the straight line from the source to the location on the detector (often approximated by the Lambert–Beer law). In other words, low-density regions (e.g. bubbles) lead to bright regions, while higher densities (e.g. particles, water) lead to dark regions in the image obtained by the detector. As before, this modality avoids both the optical access and fluid opacity issues.

The source of X-rays is generally either an X-ray tube [136–138] or a synchrotron [139–141]. The former has the advantage of being cheap and readily available as component for medical or dental equipment, but the light produced is not monochromatic and has a wider angle of emission. This wider angle can also be useful if larger fields-of-view are of interest. Synchrotrons produce a much more confined, intense, monochromatic beam. The monochromatic nature (i.e. a limited spectral band of the photons) can be preferential for quantitative measurements, as it eliminates the variation in the absorption coefficient for various parts of the spectrum (‘beam hardening’ [137]). However, synchrotrons are special facilities, and the available beam time is generally limited and/or expensive.

Detectors can be conventional (high-speed) cameras equipped with a scintillator plate (which converts X-rays to visible light) [137, 139, 141] or dedicated detectors [136, 138, 142, 143]. Naturally, strict safety rules need to be implemented when operating X-ray sources.

**Fig. 11:**
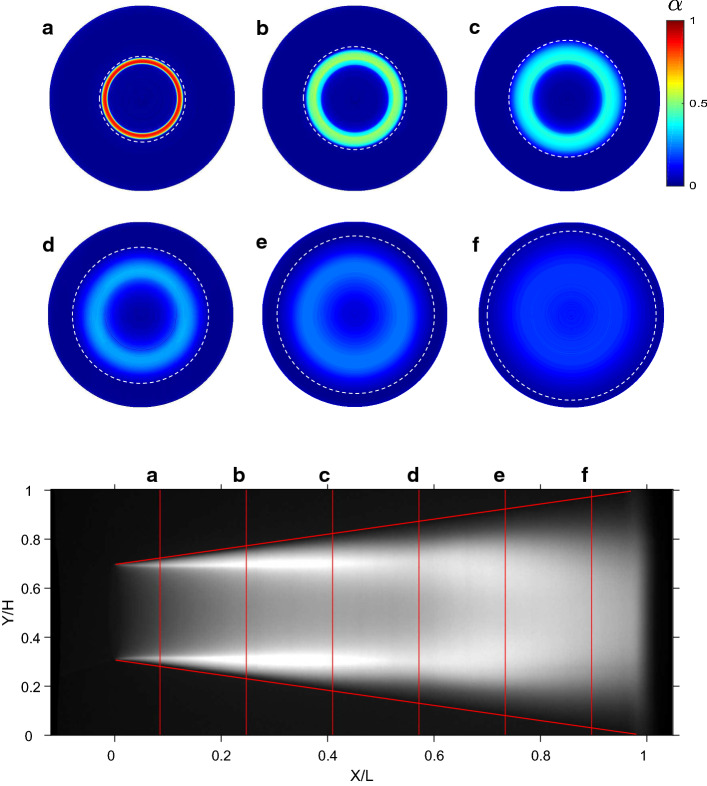
Void fraction in a cavitating venturi, obtained using X-ray computed tomography. The cross sections **a**–**f** are taken at various downstream positions. Reproduced from Jahangir et al. [138] with permission ©2019 Elsevier

An example of an X-ray tomography experiment is shown in Fig. 10, aimed at characterising the partial cavitation process in a venturi; note that this is the same geometry as the MRV example shown in Fig. 9. The X-ray result—in this case the local mean void fraction—is shown in Fig. 11.

As X-ray imaging works because of local differences in density, it is especially suitable to image flows with a large difference in density, such as air or vapour in water. It has therefore found use in, for example, the study of cavitation, but also in the field of fluidised beds [142, 143, 145]. Examples of cavitation studies include 1D measurements of the flow over a wedge [136], time-resolved 2D measurements with a similar wedge [137, 146], high-speed visualisations in a nozzle [141], and 3D measurements in a venturi [138]. In all these experiments, the local void fraction was the main goal, and often the result is qualitative [141], i.e. the technique is used as a powerful visualisation technique. To make the results for the void fraction quantitative, a careful calibration is required. This is often done using a two-point calibration: the geometry is measured when it is completely filled with water and when it is completely filled with air.^2^ A more accurate approach is the use of calibration targets, such as hollow plastic cylinders with a known diameter. These are placed in the water-filled geometry and create a known, artificial void fraction [138] to obtain a more accurate calibration curve. This is especially important for flows that have low expected void fractions, for which the two-point calibration may lead to inaccurate results.

### Computed tomography

As X-ray imaging is a shadowgraphy technique, the information that is obtained is the cumulative effect along the path between source and detector. This means that the 3D flow information is collapsed onto a single 2D plane. Most experiments therefore use a quasi-2D geometry [137, 146] or refrain from discussing 3D effects. The actual 3D spatial distribution can be retrieved using a tomographic reconstruction. In this ‘computed tomography’ (CT) technique, an inverse problem is solved to reconstruct a three-dimensional distribution, based on various projections from different angles. This can be achieved using separate detectors [145], but naturally this increases the costs of the facility. Alternatively, a single source and detector can be rotated around the field of view—the approach used in a ‘CT scan’ in a hospital. This can only provide a time-averaged end result, as the rotation is generally slow compared to the timescales of the flow of interest. Nevertheless, fast, specialised systems have been developed to obtain instantaneous 3D data [143]. An alternative can be to rotate the flow/experiment itself (if feasible) and to leave the source and detector stationary [140, 147]. Finally, if only time-averaged information is desired in an axisymmetric flow, a single view is sufficient to reconstruct the 3D result from a single X-ray projection [138].

### X-ray velocimetry

X-ray imaging requires relatively long exposures when low-power X-ray tubes are used. Furthermore, it is not trivial to construct fast mechanical shutters to obtain a pulsed illumination [148, 149]. This becomes more difficult with increasing beam diameters, such as those of X-ray tubes. Therefore, the transition from visualisation to measurement (based on consecutive image pairs) is not easy.

Early examples are the tracking of a ‘bolus’ of contrast fluid in blood vessels [150], the tracking of individual bubbles [139, 151] or particles [152] in a bubble column. PIV algorithms have been applied to X-ray images [153, 154]. Note that most of the studies simply use the projected image, so that it is difficult to interpret the obtained 2D velocity field. Fouras et al. aimed to reconstruct the true 3D field from the projected result using a deconvolution approach [155]. By utilising various projections, they demonstrated that the time-averaged, 3D flow field can be reconstructed [140].

**Table 1:** Overview of measurement techniques

Technique	What is measured?	Pros	Cons
Fluorescent tracers	Velocity of dispersed (S/L) and/or cont. phase (L/G)	Simple extension from conventional methods	Only effective in dilute systems, tracer material relatively expensive
Refractive index matching	Velocity of dispersed (S/L) and/or cont. phase (L)	No restrictions on volume fraction	Limited number of suitable fluids (parameter space)
Optical coherence tomography	Velocity of dispersed phase, relatively slow flows only	Superior resolution (micrometres)	Small penetration depth and FOV (mm)
Ballistic imaging, SLIPI	Visualisation, droplet sizing	Processing very similar to normal imaging	Complicated optical set-up
ECT	2D/3D volume fraction/phase distribution	Relatively cheap, fast	Low resolution ($\gg$1 mm)
Ultrasound	Velocity of dispersed phase (resolution $\approx$ 1 mm)	Relatively cheap, temporal resolution high	Only suitable for liquids as continuous phase
MRI/MRV	Volume fraction (qualitative), time-averaged 3D velocity (resolution $\approx$ 0.5 mm)	Can measure despite presence of gas	Expensive, material restrictions, slow
X-ray (CT)	Density/phase distribution (2D, or 3D via computed tomography)	No limitations on materials, high resolution possible (synchrotron)	Expensive, strict safety restrictions

As conventional tracer material (e.g. for PIV measurements) generally has a density close to the surrounding fluid, it will not be visible in X-ray images. Therefore, specialised tracer particles have been manufactured. For example, Seeger et al. created particles that contained a dense ‘radio-opaque’ core, surrounded by a low-density foam. This resulted in a particle with a distinct core, while maintaining neutrally buoyant characteristics [152]. Park et al. demonstrated that small $\text {CO}_2$ bubbles could act as (bio-compatible) tracers for in vivo blood flow measurements [156]. A similar approach was reported by Dubsky et al., who use contrast bubbles—intended for ultrasound imaging—to enhance the quality of their X-ray images [140].

## Summary and outlook

Dispersed multiphase flows, with their inherent turbidity, have long eluded detailed characterisation using conventional techniques. Issues such as cross-talk, a decrease in signal-to-noise ratio, and data drop-out become particularly relevant while attempting to measure in these flows. By various adaptations, the applicability of the conventional optical techniques has been stretched to higher void fractions. This was achieved by, for example, Structured Illumination and the use of fluorescent tracer particles. However, these optical techniques will remain useful only for relatively dilute suspensions. If the experiment allows for it, refractive index matching can eliminate this void fraction boundary.

In the last decade or so, many non-optical measurement techniques that rose to prominence in the clinical setting have crossed over to fluid mechanics laboratories, and they bring with them the promise of access to even the most densely laden systems. After a period of mostly development and ‘proof-of-principle’ experiments, the community is now ready to reap the benefits and obtain detailed flow information that alluded us so far.

Each of the techniques reviewed here has its unique characteristics and there is not a single ‘silver bullet’ that works for each opaque flow problem. Considerations of spatial and temporal resolution needed, costs, and accessibility all play a role in the choice of the most appropriate technique. In Table 1 a brief summary of the main characteristics is given. Several of the techniques mentioned will likely remain rather niche: it is expected that only a handful of research groups will have access to dedicated hardware needed for MRI or X-ray imaging. This is in contrast to the prevalence of optical measurement systems that are present in virtually all laboratories. As most techniques are available at medical centres, most of the work will likely be done in close collaboration with radiology departments. Of the techniques that are reviewed here, ECT and ultrasound-based techniques are the most accessible ones.

### Future perspectives

While the aforementioned collaboration with medical centres will provide easy access to state-of-the-art facilities, it also highlights a potential pitfall: it should be stressed that the use cases are obviously different for clinical work and fluid mechanics research. Both the hardware and software are developed for specific demands (anatomical dimensions, real-time flow measurements, image enhancement to distinguish anatomical structures, etc.). The hardware is also constructed to meet strict regulations as it needs to be safe to use on patients. This limits, for instance, the maximum power that can be used to create ultrasound images. Furthermore, it leads to ‘closed’ hardware, where extensions cannot be implemented. Developing custom hardware for flow measurement is possible in principle [143, 157], but likely beyond the capabilities or interests of most fluid mechanics laboratories.

In contrast to the hardware aspects, there is a huge potential on the software side. While earlier studies had to rely on the proprietary software used for clinical studies, it is now more and more feasible to run custom software on standard hardware. This has two main advantages: (i) advanced acquisition protocols can be implemented, and (ii) raw data are available. Examples of the former include, for example, interleaved imaging to allow for the measurement of fast flows in echography [87], or the use of the ‘SYNC SPI’ sequence for fast flows in MRV [115]. Access to raw data is essential to maximise the accuracy of flow measurement. It also allows researchers to post-process the same raw data with different algorithms to see which one performs better. Obviously, this is not feasible when the device only provides processed data—the latter being a direct consequence of the need for clinicians to have robust (near) real-time data. In fluid mechanics research, research strategies are generally based on offline processing.

In the last decade, a considerable number of ‘proof-of-concept’ studies that demonstrate the feasibility of a new technique have been reported—in fact most of the studies referenced here fall within this category. Unfortunately, not all of them compare the results that are obtained to reference data. This is in part due to the fact that little of such data is often available. A notorious case is that of cardiovascular flow, where it is nearly impossible to perform two sets of measurements with different techniques under the exact same conditions. Regardless of the application area, there should be an effort to at least perform a basic check of the data. One easy example is checking if mass is conserved, by integrating the velocity profile at various downstream locations [115]. An alternative can be to link pressure drop measurements with the wall shear stress to evaluate whether velocity gradients at the wall are resolved. Finally, evaluating data scatter (i.e. spatial or temporal variation) in a flow that is expected to be laminar can provide an estimate of measurement errors. An honest assessment of the measurement accuracy and/or limitations will not only promote the uptake of the techniques, but also help in selecting the best data acquisition and processing strategies.

An important strategy in pushing the state of measurement techniques forward is to use well-described benchmark cases. For single-phase flow, fully developed pipe flow is the most common one. For laminar flow, we have the exact solution (‘Poiseuille’), and for turbulent flow we have an abundance of trusted empirical data [158]. For dispersed multiphase flows, these benchmarks have not yet been established. One approach could be to test techniques using a relatively low volume fraction and compare to single-phase results [102]. However, it is a better strategy to design specific multiphase cases. The example of the cavitating flow in a venturi (Figs. 9, 11) can be considered to be an effort to establish such a benchmark flow. By various techniques, a myriad of data is now available that *should* in theory all be reconcilable. With these benchmarks, it is possible to compare various processing strategies and evaluate the performance under specific conditions.

Despite these open questions, it has been established that the techniques reviewed here provide exciting possibilities to study dispersed multiphase flows. They are ready to be used to study new phenomena or to provide validation data for numerical simulations. They will likely push the field forward, as with these tools we can finally unveil opaque flows.

## Footnotes

1 The SLIPI image is obtained from the pair-wise differences of the three modulated images, each with a $2\pi /3$ phase shift [63]: $I_s=\sqrt{2}/3\cdot [(I_1-I_2)^2+(I_1-I_3)^2+(I_2-I_3)^2)]^{1/2}$.

2 Filling the geometry with vapour is difficult to do in practice, so the small error due to the difference in the density of water vapour and air is ignored.
